# Possible use of a H3R antagonist for the management of nonmotor symptoms in the Q175 mouse model of Huntington's disease

**DOI:** 10.1002/prp2.344

**Published:** 2017-08-22

**Authors:** Daniel S. Whittaker, Huei‐Bin Wang, Dawn H. Loh, Roger Cachope, Christopher S. Colwell

**Affiliations:** ^1^ Department of Psychiatry & Biobehavioral Sciences University of California Los Angeles California 90095‐1751; ^2^ CHDI Foundation 6080 Center Drive Suite 100 Los Angeles California 90045

**Keywords:** Circadian rhythms, GSK189254, histamine, histamine‐3 receptor, Huntington's disease, nonmotor symptoms, sleep behavior

## Abstract

Huntington's disease (HD) is an autosomal dominant, neurodegenerative disorder characterized by motor as well as nonmotor symptoms for which there is currently no cure. The Q175 mouse model of HD recapitulates many of the symptoms identified in HD patients including disruptions of the sleep/wake cycle. In this study, we sought to determine if the daily administration of the histamine‐3 receptor (H3R) antagonist/inverse agonist 6‐[(3‐cyclobutyl‐2,3,4,5‐tetrahydro‐1H‐3‐benzazepin‐7‐yl)oxy]‐N‐methyl‐3‐pyridinecarboxamide hydrochloride (GSK189254) would improve nonmotor symptoms in the Q175 line. This class of drugs acts on autoreceptors found at histaminergic synapses and results in increased levels of histamine (HA). HA is a neuromodulator whose levels vary with a daily rhythm with peak release during the active cycle and relatively lower levels during sleep. H3Rs are widely expressed in brain regions involved in cognitive processes and activation of these receptors promotes wakefulness. We administered GSK189254 nightly to homozygote and heterozygote Q175 mice for 4 weeks and confirmed that the plasma levels of the drug were elevated to a therapeutic range. We demonstrate that daily treatment with GSK189254 improved several behavioral measures in the Q175 mice including strengthening activity rhythms, cognitive performance and mood as measured by the tail suspension test. The treatment also reduced inappropriate activity during the normal sleep time. The drug treatment did not alter motor performance and coordination as measured by the challenging beam test. Our findings suggest that drugs targeting the H3R system may show benefits as cognitive enhancers in the management of HD.

**Abbreviations**
CB: challenging beam
GSK189254: 6‐[(3‐cyclobutyl‐2,3,4,5‐tetrahydro‐1H‐3‐benzazepin‐7‐yl)oxy]‐N‐methyl‐3‐pyridinecarboxamide
H3R: histamine‐3 receptor
HA: histamine
HD: Huntington's disease
KO: knockout
LD: light/dark
OF: open field
RI: resident‐intruder
SCN: suprachiasmatic nucleus
TMN: tuberomammilary nucleus
TM: T‐maze
TS: tail suspension
ZT: zeitgeber time

## Introduction

Huntington's disease patients suffer from a progressive neurodegenerative process that inflicts cognitive, psychiatric, and motor dysfunction (Margolis and Ross 2003; Kuljis et al. 2012a). HD is caused by a CAG repeat expansion within the first exon of the *huntingtin* (*Htt*) gene which produces a polyglutamine repeat that leads to protein misfolding, soluble aggregates, and inclusion bodies detected throughout the body (Saft et al. 2005; Ciammola et al. 2006). The normal function of the protein (HTT) remains unknown; however, the mutated form leads to dysfunction of a large range of cellular processes including cytoskeletal organization, protein folding, metabolism, and transcriptional activities (reviewed by Saudou and Humbert 2016). HD symptoms start at a range of ages, with an average onset at 40 years of age. Generally, the longer the CAG repeat, the earlier the age of onset and the greater the severity of the symptoms (Duyao et al. 1993; Langbehn et al. 2010). Still, even among patients with the same CAG repeat length, there is considerable range in the onset of symptoms (around a decade) and their severity (Wexler 2004; Gusella et al. 2014). This variability raises the possibility that optimal disease management can increase the health span of the HD patients.

Histaminergic neurons originate in the tuberomammilary nucleus (TMN) which is in the hypothalamus and broadly releases HA throughout the central nervous system. The release is controlled with a diurnal rhythm with high neural activity in the TMN and subsequent HA release peaking during the organism's active cycle (night time in mice). The postsynaptic effect is largely excitatory and thus HA is considered one of the main transmitters that control arousal (Haas and Panula 2003). One of the major HA outflows targets the central clock, the suprachiasmatic nucleus or SCN (Watanabe et al. 1984), and HA is a potent regulator of the circadian system (e.g., Cote and Harrington 1993; Eaton et al. 1995; Meyer et al. 1998; Jacobs et al. 2000; Parmentier et al. 2002; Abe et al. 2004; Kim et al. 2015). Commonly prescribed wake‐promoting compounds, such as methamphetamine‐related drugs or modafinil, require a functional dopaminergic system (Wisor et al. 2001). Since dopaminergic pathways are compromised in HD, H3Rs are a promising target to treat hypersomnia and H3R antagonists promote wakefulness (Lin et al. 2011). In HD patients, there is evidence that histaminergic signaling is disrupted and could contribute to disrupted rhythms in arousal in HD patients (van Wamelen et al. 2011; Shan et al. 2012). Disruptions in the sleep/wake cycle are an early part of HD symptoms seen in both patients and mouse models (e.g., Morton et al. 2005). On the basis of this literature, we hypothesized that the nightly treatment with a H3R antagonist would not only acutely increase arousal but also adjust the phase of the underlying circadian system to improve rhythmic behaviors.

Among the mouse models of HD, the heterozygote (Het) Q175 offers advantages of single copy of the mutation, genetic precision of the insertion and control of mutation copy number (Pouladi et al. 2013). These features make the Het Q175 line perhaps the most clinically relevant of the mouse lines. Prior work with this model has demonstrated that the Q175 line exhibits age‐dependent disruptions in motor performance (Heikkinen et al. 2012; Menalled et al. 2012) as well as circadian behavior (Loh et al. 2013) when compared with wild‐type controls. In this study, we sought to test the hypothesis that the daily chronic treatment with a H3R antagonist would improve the nonmotor symptoms early in the disease progression of the Q175 model. Homozygote (Hom) and Het Q175 mice received daily treatment in the early night (zeitgeber time [ZT] 13) of GSK189254 for 1 month. We evaluated the effect of the drug compared to vehicle controls on acute locomotor activity as well as on daily rhythms in activity and sleep behavior. In addition, we examined the impact of the treatment regime on the HD model's performance in open field, T‐maze, and tail suspension tests. Finally, as controls, we examined the impact of the treatment on motor performance and resident‐intruder aggression.

## Materials and Methods

All experimental protocols used in this study were approved by the University of California, Los Angeles (UCLA) Animal Research Committee. Every effort was made to minimize pain and discomfort. Experiments followed the UCLA Division of Laboratory Animal Medicine recommendations for animal use and welfare, as well as National Institutes of Health guidelines.

### Animals

A line of mice with a spontaneous expansion of the CAG repeats, the Q175 mutation (Menalled et al. 2012), was obtained from the CHDI colony of Q175 mice at the Jackson Laboratories (Bar Harbor, Maine). We obtained cohorts of male 9–month‐old Q175 Het mice with CAG repeats averaging 191 ± 4, and male 4‐month‐old Q175 Hom mice with CAG repeats averaging 183 ± 9. Mice were housed in standard mouse cages inside custom cabinets in a light‐tight setting allowing continuous sleep behavior recording. All mice were held in a light/dark (LD) cycle of 12 h of light followed by 12 h of dark (LD 12:12) with an average 350 lux white LED light (HitLights LED, Baton Rouge, LA) during light periods. Under LD 12:12 conditions, the time of lights‐on defines ZT 0, whereas the time of lights‐off is defined as ZT 12. All animals received rodent chow and water ad libitum.

### Drug treatment

GSK189254 has a reported oral half‐life of 2.5 hrs and is highly brain penetrant. Two hrs after dosing by oral gavage, 1.0 and 3.0 mg/kg are reported to result in serum levels of 115 and 239 ng/mL and 80 ± 9 and 90 ± 2% receptor occupancy, respectively (Medhurst et al. 2007; Wilson et al. 2013). While prior work has used oral gavage to deliver the drug, we found that this drug delivery method disrupted activity rhythms in C57 mice (data not shown). Therefore, in this study, we placed the vehicle or drug into condensed milk. GSK189254 was provided in lyophilized form by the CHDI Foundation. The vial was maintained in an airtight bag inside a light‐tight box in a 4°C fridge. The drug compound was prepared weekly for administration by grinding the measured antagonist into a fine powder using mortar and pestle, then bringing into suspension by slowly adding 100 *μ*L of 0.5% methylcellulose in saline and mixing for five min. This was then mixed incrementally into 10 g condensed milk (NESTLÉ, Wilkes‐Barre, PA) for 10 min followed by 15 min of sonication. Amounts of GSK189254 used depended on the final drug concentration needed in order to generate 0.3, 1.0, or 3.0 mg/kg dosing. Following sonication, final compound formulations were immediately aliquoted into prelabeled 1.5 mL tubes, wrapped with parafilm, boxed, and stored in a 4°C fridge until used. Vehicle treatment was similarly prepared with 100 *μ*L 0.5% methylcellulose in saline and 10 g condensed milk.

The compound was administered daily at ZT 13. Aliquots were sonicated for 10 min, with the condensed milk level held below the sonicator water level. Mouse weights were consulted to determine the amount of compound to administer to each mouse. 10 mg of compound or vehicle for every gram of body weight was weighed into preweighed dishes, one per mouse, using a microbalance. Under dim red light (3‐5 lux), the dishes were placed in the corresponding cages and collected 20 min later. Each preweighed dish was re‐weighed and differences were recorded to ensure all compound or vehicle was consumed.

As an initial control experiment to ensure that our drug administration method was successful, mice of each genotype were randomly placed into one of the four treatment groups: vehicle, and 0.3, 1.0, or 3.0 mg/kg GSK189254. After drug was administered for 4 weeks, serum was collected 1 h posttreatment. Blood was collected (about 0.7–0.9 mL per animal) via cheek puncture into microvette EDTA tubes (Sarstedt, Nümbrecht, Germany). Tubes were gently inverted a few times and placed immediately on wet ice. Within 1 h following collection, samples were centrifuged at 660 g for 15 min at 4°C. Using a new pipet per sample, the supernatant was collected (0.1–0.25 mL) into prelabeled Eppendorf tubes (Fisher Scientific, Hampton, NH). Tube lids were covered with parafilm and immediately stored at −80°C until shipment. Multiple samples of each formulation prepared were also collected and stored. Measurements of GSK189254 were made with liquid chromatography‐mass spectrometry by Discovery ‐ Charles River (Essex, UK). Vehicle controls were not detectable. We subsequently used 3.0 mg/kg for the remainder of the study. Body weights were recorded weekly.

### Experimental design

Initially mice were habituated to the LD cycle and housing conditions, we then measured diurnal rhythms in activity and sleep over a 2‐week period from Hom and Het Q175 mice. The performance of the mice on open field, T‐maze, tail suspension, challenging beam, and resident‐intruder tests was assessed in the early night (ZT 14‐17) under dim red light (3‐5 lux) under these baseline conditions. Next, the mice were randomly divided into two groups with one group treated with GSK189254 and one group receiving the vehicle control. During the treatment period, mice were given access to a small dish with vehicle or vehicle + drug placed in their cage for 20 min at ZT 13. After 2 weeks of treatment, rhythms in activity and sleep were measured. After 4 weeks of treatment, behavioral tests were repeated while the mice were still receiving vehicle or drug. Again, all tests were performed under dim red light (3‐5 lux) in the middle of the night: during ZT 14‐17 to coincide with peak drug activity. This design allowed us to both compare the behavioral measures from the drug‐ and vehicle‐treated groups at the end of treatment but also to examine the performance of the same animals before and after treatment. All nonautomated behavioral measurements were carried out by two independent observers who were masked as to the experimental condition and the results averaged.

The sample size of 10 mice per group was determined by both our empirical experience with locomotor activity measures in the Q175 mice and a statistical analysis (SigmaPlot, v13) that assumed a power of 0.8 and an alpha of 0.05. For the liquid chromatography‐mass spectrometry measurements of GSK189254, one of the Het Q175 samples was corrupted during collection or shipment and was excluded from the analysis. In addition, a recording error was made during the T‐maze tests with one of the vehicle‐treated Het Q175 mice so that the sample size was 9 rather than 10 mice.

### Video measurement of immobility‐defined sleep

Immobility‐defined sleep was determined as described previously (Loh et al. 2013). Mice were housed in see‐through plastic cages containing bedding, but without the addition of nesting material. Video capture of a side‐on view of each cage was obtained, and was not obstructed by the top mounted food bin or water bottle. Cages were under constant infrared LED lighting. Video was captured using infrared surveillance cameras (DTV Electronics, Inc., San Leandro, CA) equipped with IR850 infrared filters (Neewer Technology Ltd., Guangdong, China) and connected to video‐capture cards (Adlink Technology Inc., Irvine, CA). ANY‐maze software (Stoelting Co., Wood Dale, IL) was used to track the animals. Immobility detection was set to 95% of the area of the animal immobile for 40 sec, as was previously determined to have 99% correlation with simultaneous EEG/EMG defined sleep (Pack et al. 2007; Fisher et al. 2012). We recorded video continuously, except when administering treatments, performing testing, and changing cages. For each time period analyzed, we used five consecutive days in the period for sleep analysis and three consecutive days for analysis of bouts. Immobility‐defined sleep was determined in 1‐min bins and total sleep was determined by summing immobility durations. A sleep bout was defined as a duration of continuous immobility (maximum gap: 1 min; threshold: 3 counts/min), and the number of bouts and the average duration of bouts were determined for both day and night using ClockLab software (Actimetrics, Wilmette, IL). An average waveform of hourly sleep over 5 days was produced per genotype and per age and treatment group for the purpose of graphical display.

### Cage activity recording

Locomotor activity recording was performed using passive IR sensors (Honeywell International, Inc., Morris Plains, NJ) and recorded with a data acquisition system obtained from Mini Mitter Co. (Bend, OR). Data were analyzed using the El Temps (A. Diez‐Noguera, Barcelona, Spain) and ClockLab programs. We recorded continuously, except when performing behavior testing, and changing cages on alternate Mondays. For each time period analyzed, cage activity was recorded in 3‐min bins, and 10 days of data was averaged for analysis. The data were analyzed in El Temps to determine the period and rhythmic strength (Kudo et al. 2011; Loh et al. 2013). Major activity duration (alpha, *α*) was determined in El Temps by the duration of continuous activity over the threshold of the mean using an averaged waveform. Onset variability was determined by calculating the daily variation in onset over 10 days of activity using the Clocklab program. Acute activity in response to treatment was also assessed by obtaining activity in El Temps for one, two, and three hrs post administration and calculating the fraction of total daily activity represented in each respective hour and for the 3‐h period. For acute activity, the last day of untreated acclimation was compared to the first day of administration following habituation.

### Open field test

The OF test was used to assess anxiety‐like behavior (Prut and Belzung 2003; Bailey and Crawley 2009; Southwell et al. 2009) as well as exploratory behavior and spontaneous locomotor activity (Rothe et al. 2015). Animals were individually placed in a plastic arena with opaque walls (47 cm wide × 40 cm long × 30 cm tall). The experimenter moved out of the testing area after placement of animals. OF activity was recorded for 10 min by a ceiling‐mounted infrared equipped 800TVL dome video camera (101 AudioVideo Inc., Sunnyvale, CA). The tests were performed under dim red light (3‐5 lux). ANY‐maze software was used to code the videos. Anxiety‐like behavior was scored for time spent in the center versus the peripheral zone of the testing arena. The central 25% of the arena was designated as the center zone. To assess exploratory behavior and spontaneous locomotor activity, speed and total distance travelled over 10 min were scored using ANY‐maze software.

### Tail suspension test

The TS test was employed to develop an index of behavioral despair based upon the duration of immobility, where longer durations of immobility imply a greater degree of behavioral despair (Cryan et al. 2005; Juszczak et al. 2006). The method used was similar to that described previously (Can et al. 2011). Briefly, tape is applied to the tails of mice and they are suspended 15 cm below a bar and in front of a white background for six min. The tests were video recorded during the early night under dim red light (3‐5 lux) and manually scored post hoc for their mobility time by two independent investigators. Mobility time is subtracted from total time to generate immobility time.

### T‐maze test

The TM test was performed to assess cognitive ability (Deacon and Rawlins 2006) in the mice. The TM (UCLA Psychology Department shop, Los Angeles, CA) was mounted on a tripod and consists of two arms perpendicular to one another forming the T‐maze. Each arm of the TM is 290 mm and the “T” junction is 80 mm × 80 mm. All walls of the TM are 145 mm tall and opaque. No habituation to the maze is used, as it is the novelty of the maze that drives the spontaneous alternation exploratory behavior. Mice were tested in their active phase and were not food‐deprived prior to testing. Dim red lighting (3‐5 lux) was used and placed directly over the center of the “T” to avoid shadows on the starting arm. Prior to the testing, mice were acclimated to the testing room. At the start of each test, a well‐handled mouse is placed at the base of the TM on the starting platform and the experimenter steps straight back away from the maze. The mouse chooses an arm at the “T” junction and is blocked in the arm once all four feet enter the arm. The mouse is retained for 20–30 sec in the arm before starting the next trial. Each mouse undergoes 10 trials. Scoring is performed by recording the choice each trial.

### Challenging beam test

The CB test for motor performance and coordination was performed as previously described (Fleming et al. 2004, 2013; Loh et al. 2013). Briefly, animals were trained to walk across an elevated beam (Plastic Zone Inc., Tarzana, CA) ending in their home cage. The beam consists of progressively narrowing 253 mm lengths, with widths of 33 mm, 24 mm, 18 mm, and 6 mm. Training and testing were conducted during the early night under dim red lighting (3‐5 lux). Training was conducted for two consecutive days with five beam crossings per mouse each day. On the third day, when testing was conducted, raised 19‐gauge mesh wire grids (Ace Hardware, Oak Brook, IL) with 10 mm × 10 mm spacing were overlaid onto the beams. Mice were video recorded from the side as they crossed the gridded beams. Videos were scored post hoc by two independent investigators for the number of step errors made per beam. Errors were counted when the mouse was facing and moving forward. A step was considered an error when any foot passed below the grid and more than half‐way to the beam. Counting began when the mouse had all paws on the grid and made its first step forward. Counting ended when the mouse stepped with either forelimb off the end of the final beam grid. Scores were averaged for each beam segment for the five trials per mouse.

### Resident‐intruder test

The resident‐intruder test was performed as described (Koolhaas et al. 2013) to assess the aggression in untreated mice at baseline and in response to the treatment. The “resident” mice (Q175) were acclimated in their home cage for five min before the tests began. Unfamiliar C57 WT mice with lighter body weights were selected as the “intruder” mice. The test started when the intruder mouse was introduced to the resident's cage and preceded for five min. The tests were video recorded during the early night under dim red light (3‐5 lux) and manually scored post hoc for aggressive behaviors by two independent investigators. Exploration time before first attack, total time of attacks, and the duration of the longest attack were recorded. The scores from the two scorers were averaged per mouse.

### Statistical analysis

Statistical analysis was performed using SigmaPlot (v. 13). Datasets were examined for normality (Shapiro–Wilk test) and equal variance (Brown–Forsythe test). The values from the groups were analyzed using a two‐way or one‐way analysis of variance (ANOVA). Pairwise multiple comparison procedures were made using the Holm–Sidak method. In the cases where the normality or equal variance assumptions were not met, a Kruskal–Wallis one‐way ANOVA on ranks were used. Finally, paired *t*‐tests were used to determine if the drug treatment altered the performance of the mice before and after treatment. Statistical significance was defined by *P* < 0.05 in all analyses.

## Results

### Oral administration of 3.0 mg/kg was an effective dose of GSK189254

**Figure 1:**
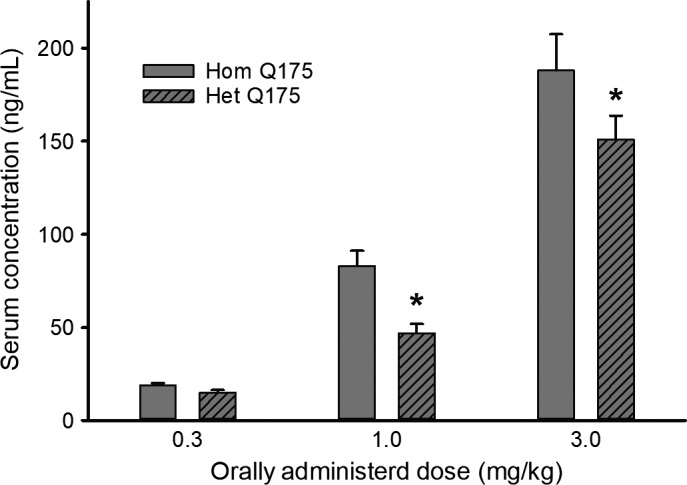
NOTE y‐axis label “S” in serum (top of letter is smudged/blurred) Serum was collected 1 h after oral administration of drug in condensed milk. Measurements made with liquid chromatography‐mass spectrometry by Discovery ‐ Charles River (Essex, UK). Vehicle controls were not detectable. The data were analyzed with a two‐way ANOVA with dose and genotype as factors. Values are mean ± SEM in this and remainder of the figures. Asterisk represents significant genotypic differences (*P* < 0.05). The serum concentrations for the 1.0 and 3.0 mg/kg treatments were significantly (*P *<* *0.05) higher than those measured after the 0.3 mg/kg treatment.

In our first experiment, we sought to determine what concentration of GSK189254 would be sufficient to increase serum levels of the drug to biologically meaningful levels. Hom and Het Q175 mice (*n* = 10 per group) were randomly placed into one of the four groups: vehicle, and 0.3, 1.0, 3.0 mg/kg GSK189254. After drug or vehicle was administered for 4 weeks, serum was collected between ZT 14‐15 and levels of GSK189254 determined by liquid chromatography‐mass spectrometry. The resulting measurements were analyzed with a two‐way ANOVA with dose and genotype as factors. There was a significant effect of dose (*F *=* *87.846, *P *<* *0.001) with 3.0 mg/kg moving the serum levels well above our target of 100 ng/mL (Fig. 1). Interestingly, at both the 1.0 and 3.0 mg/kg dose, the serum levels were lower (*P *<* *0.05) in the Het Q175 than in the Hom Q175 (Fig. 1). Since the effective concentration varied between the genotypes, we analyzed the findings from the two genotypes separately for the remainder of the study.

Chronic GSK189254 produced short‐term increases in activity without altering the total activity over 24 h.

**Figure 2:**
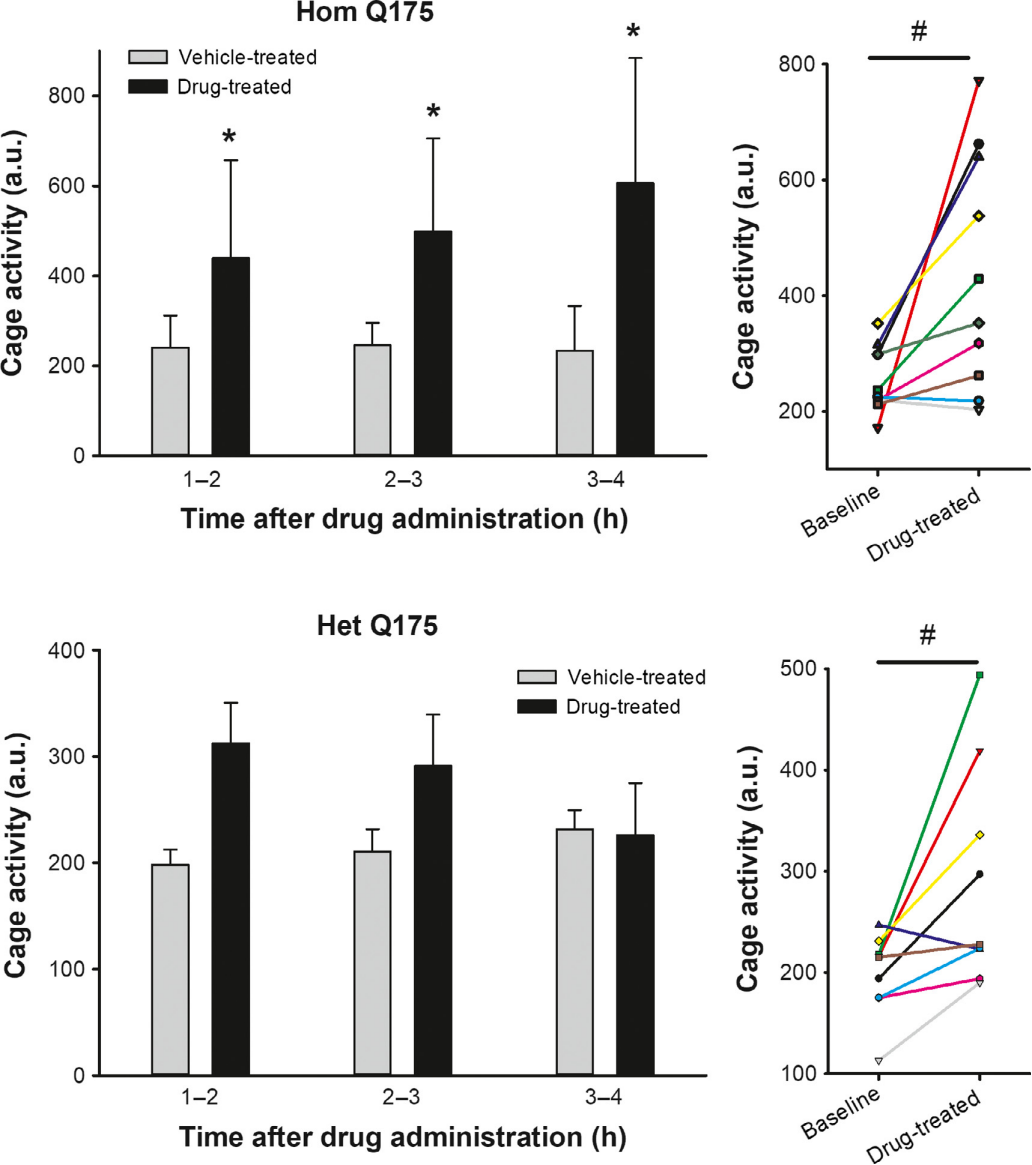
Hom Q175 (top of letter “H” and numbers smudged/blurred) and y‐axis “C” in cage activity (Hom bar graph) also same. The acute oral administration of GSK189254 (3.0 mg/kg) significantly increased total cage activity measured by an IR sensor in Hom (top) and Het (bottom) Q175. Left panels illustrate the activity for the 3 h following treatment. After 4 h, activity was no longer elevated compared to vehicle controls in either genotype. Two‐way ANOVA was used to analyze the activity levels with time and treatment as factors. Asterisks represent significant differences due to drug treatment compared to vehicle‐treated controls (*P *<* *0.05). Right panels show the values from individual animals at the same ZT on the last day of untreated acclimation compared to the first day of administration 1 h after treatment. Paired *t*‐test found significant (*P *<* *0.05) impact of drug treatment on the animal's activity levels shown by a # symbol. Most, but not all, mice exhibited a significant increase in activity as a result of drug treatment.

The acute impact of the oral administration of GSK189254 (3.0 mg/kg) was assessed by monitoring the amount of total cage activity measured by IR sensor in Hom and Het Q175 at one, two, and three hrs after treatment (Fig. 2; two‐way repeated measures ANOVA with time and treatment as factors). Compared to vehicle‐treated controls, drug treatment significantly increased (*F *=* *15.485, *P *<* *0.001) cage activity of the Hom Q175 and a multiple comparison procedure indicated that increase was seen (*P *<* *0.05) at each of the time points following treatment. In contrast, compared to vehicle‐treated controls, drug treatment did not alter the activity of the Het Q175 (*F *=* *4.075, *P *=* *0.06). A comparison of the same animals at the same ZT on the last day of baseline untreated acclimation and on the first day of administration after habituation (Fig. 2; Paired *t*‐test) indicated that the drug treatment increased activity in both the Hom (*t *= −2.984, *P *=* *0.015) and Het (*t *= −2.854; *P *=* *0.021) Q175 when measured 1 h after treatment. Most Hom Q175 (8/10) and all the Het Q175 (9/9) mice exhibited increased activity levels as measured 1 h after treatment when compared with baseline. As measured over the full 24‐h cycle, the treatment did not significantly alter activity levels in either the Hom (*F *=* *1.533, *P *=* *0.223) or Het (*F *=* *1.838, *P *=* *0.158) Q175. There was a trend toward lower overall activity in both the Hom (baseline: 157 ± 18 au/h; vehicle: 156 ± 13 au/h; drug: 130 ± 8 au/h) and Het (baseline: 174 ± 18 au/h; vehicle: 169 ± 18 au/h; drug: 133 ± 11 au/h) Q175. For this and other behavioral measures, the WT data are shown in Table 1 for comparison. Overall, the drug produced an acute increase in activity that lasted over three hrs in the Hom Q175 line.

**Table 1:** Selected behavioral values for WT control mice. While not part of the same study, the WT data were obtained using the same equipment and protocols as those used with the Q175 mice. The Het Q175 mice were tested at 9 months, whereas the Hom Q175 at 6 months of age

Task	WT (6 months)	WT (9 months)
Activity (au/h)	218 ± 28	275 ± 32
Power (%V)	39 ± 3	30 ± 1
Activity in light (%)	20 ± 1	20 ± 1
Onset variability (min)	11 ± 1	26 ± 2
Sleep in day (min)	474 ± 10	534 ± 12
Sleep in night (min)	167 ± 14	174 ± 14
Sleep bouts in day (#)	23 ± 1	26 ± 2
Sleep bouts in night (#)	30 ± 4	44 ± 3
OF distance (m)	43 ± 2	40 ± 2
OF speed (m/s)	0.07 ± 0.001	0.07 ± 0.001
OF center time (%)	14 ± 1	21 ± 1
TM spontaneous alternation (#)	5.3 ± 0.6	5.6 ± 0.5
RI aggressive interactions	20%	12%
CB errors (#)	4.4 ± 0.5	5.5 ± 0.3

### Chronic GSK189254 strengthened daily activity rhythms

**Figure 3:**
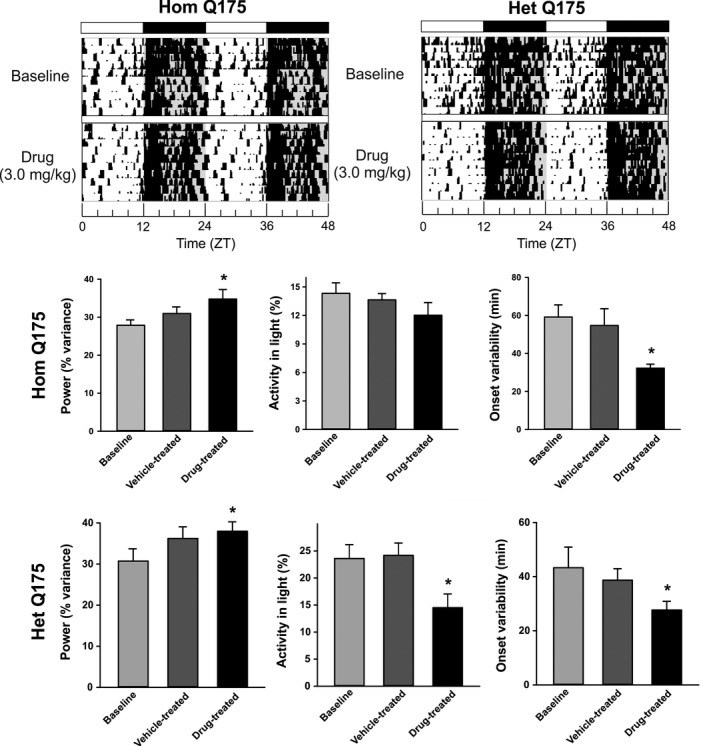
Chronic administration of GSK189254 improved some aspects of the daily rhythm in cage locomotor activity in Hom and Het Q175. Top panels show representative examples of cage locomotor activity at baseline and during drug treatment. Drug treatment improved some of the key measurements of activity rhythms in Hom (middle panels) and Het (bottom panels) Q175. One‐way ANOVA was used to analyze the group values. Asterisks represent significant differences due to drug treatment compared to vehicle‐treated controls (*P *<* *0.05).

Next, we examined the impact of the drug on activity rhythms of the Q175 mice recorded over 10 days. Compared to vehicle‐treated mice, administration of GSK189254 improved several measures of the daily rhythm in locomotor activity in Hom and Het Q175 (Fig. 3; one‐way ANOVA). In the Hom Q175, the drug treatment increased the power (*F *=* *3.576, *P *=* *0.023) and reduced the variability in the onset of the nightly activity bout (*H *=* *14.475, *P *=* *0.002). In the Het Q175, the drug treatment increased the power (*F *=* *4.663, *P *=* *0.007) as well as reduced the variability in the onset of the nightly activity bout (*F *=* *5.233, *P *=* *0.004) and inappropriate daytime activity (*F *=* *8.917, *P *<* *0.001). A comparison of the same animals before and after treatment (paired *t*‐test) indicated that the drug treatment also significantly improved power and precision of the activity rhythms of both genotypes and the % activity in the light in the Het Q175 (data not shown). Thus, GSK189254 treatment improved activity rhythms in both Het and Hom Q175 lines.

### Chronic GSK189254 altered the daily pattern of sleep behavior

**Figure 4:**
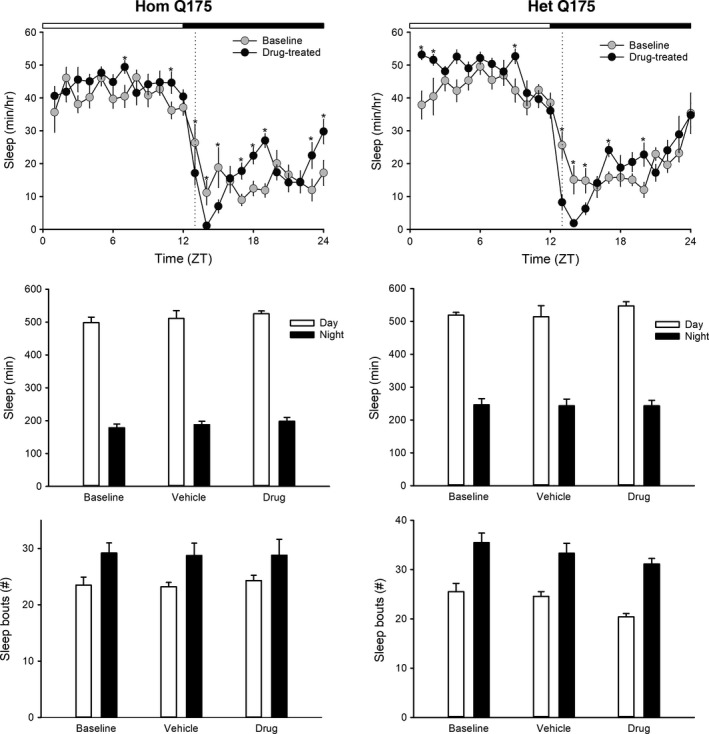
Chronic administration of GSK189254 alters the temporal pattern of sleep behavior in Q175 mice. Video recording in combination with automated mouse tracking analysis software was used to measure immobility‐defined sleep. Top panels show running averages (1‐h window) of immobility‐defined sleep in Hom Q175 (left) and Het Q175 (right) before and during drug treatment. The time of daily drug administration is shown by the dashed line. When examined by day and night (12‐h bins), the drug treatment did not alter sleep duration (middle panels) or sleep fragmentation (bottom panels). One‐way ANOVA was used to analyze the group values but none of these values were significantly different.

We also examined the impact of the drug on rhythms in sleep behavior measured over 1‐week periods with video recording in combination with an automated mouse tracking analysis software system (Li et al. 2015; Wang et al. 2017). In both Hom and Het Q175, the treatment with GSK189254 produced an acute decrease in sleep which lasted for several hrs (Fig. 4, top panels). A 2‐way ANOVA was used to analyze the 1‐h bins and found significant effects of treatment (Hom: *F* = 9.176, *P* = 0.003; Het: *F* = 7.302, *P* = 0.007) and time (Hom: *F *=* *42.402, *P *<* *0.001; Het: *F *=* *51.890, *P *<* *0.001). However, the treatment did not alter the total amount of sleep (Fig. 4, middle panels) or the number of sleep bouts (Fig. 4, bottom panels) in the day or night in either of the mutant lines. A comparison of the same animals before and after treatment (paired *t*‐test) indicated that the drug treatment produced significant increases in the duration (*t *= −2.742, *P *=* *0.023) and decreased the number of sleep bouts (*t *=* *3.425, *P *=* *0.007) in the Het Q175 in the day (data not shown). The rest of the values were not significantly different. Thus, GSK189254 treatment produced changes in the temporal pattern of sleep behavior but did not cause sleep deprivation in either the Hom or Het Q175 lines.

### Chronic GSK189254 improved performance on OF and TM tests without increasing anxiety‐like behavior

**Figure 5:**
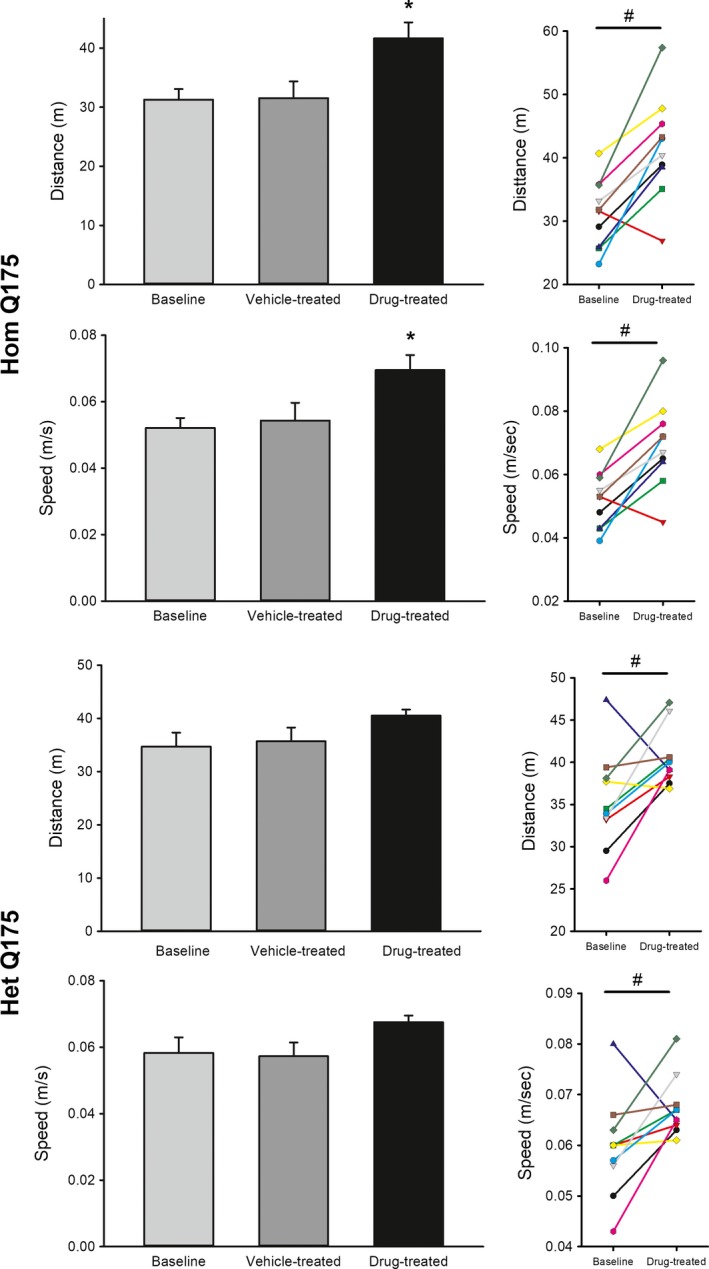
Chronic administration of GSK189254 improved exploratory behavior as measured by the open field test in Hom and Het Q175 mice. One‐way ANOVA was used to analyze the group values. Asterisks represent significant differences due to drug treatment compared to vehicle‐treated controls (*P *<* *0.05). Paired *t*‐test found significant (*P *<* *0.05) impact of drug treatment on the animal's activity levels before and after treatment, shown by a # symbol. Most, but not all, mice exhibited a significant increase in exploratory activity as a result of drug treatment.

We examined the impact of the drug on two commonly used tests that are both dependent upon activity measures. Administration of GSK189254 improved exploratory behavior as measured in the OF test (Fig. 5). Compared to vehicle‐treated controls, drug treatment significantly increased both the distance (*F *=* *7.279, *P *=* *0.001) and speed of movement (*F *=* *6.240, *P *=* *0.002) in the Hom Q175. A comparison of the same animals before and after treatment (paired *t*‐test) indicated that the drug treatment also significantly improved distance (*t *= −4.532, *P *=* *0.001) and speed (*t *= −4.485, *P *=* *0.001) of the Hom Q175. The drug treatment did not change the amount of time the mice spent in the center of the OF arena (baseline: 9 ± 1%; vehicle: 14 ± 2%; drug: 13 ± 2%; *F *=* *3.001, *P *=* *0.067).

The drug‐treatment had a less robust impact on the OF behavior in the Het Q175. The GSK189254 treatment did not significantly increase posttreatment values for distance (*H *=* *5.707, *P *=* *0.127) and speed of movement (*H *=* *7.169, *P *=* *0.057). A comparison of the same animals before and after treatment (paired *t*‐test) indicated that the drug treatment did significantly improved distance (*t *= −2.542, *P* = 0.032) and speed (*t *= −2.335; *P* = 0.044) in the Het Q175 (Fig. 5). The treatment did not alter the amount of time the mice spent in the center of the OF arena (baseline: 17 ± 2%; vehicle: 16 ± 2%; drug: 17 ± 3%; *H *=* *0.018; *P *=* *0.991).

**Figure 6:**
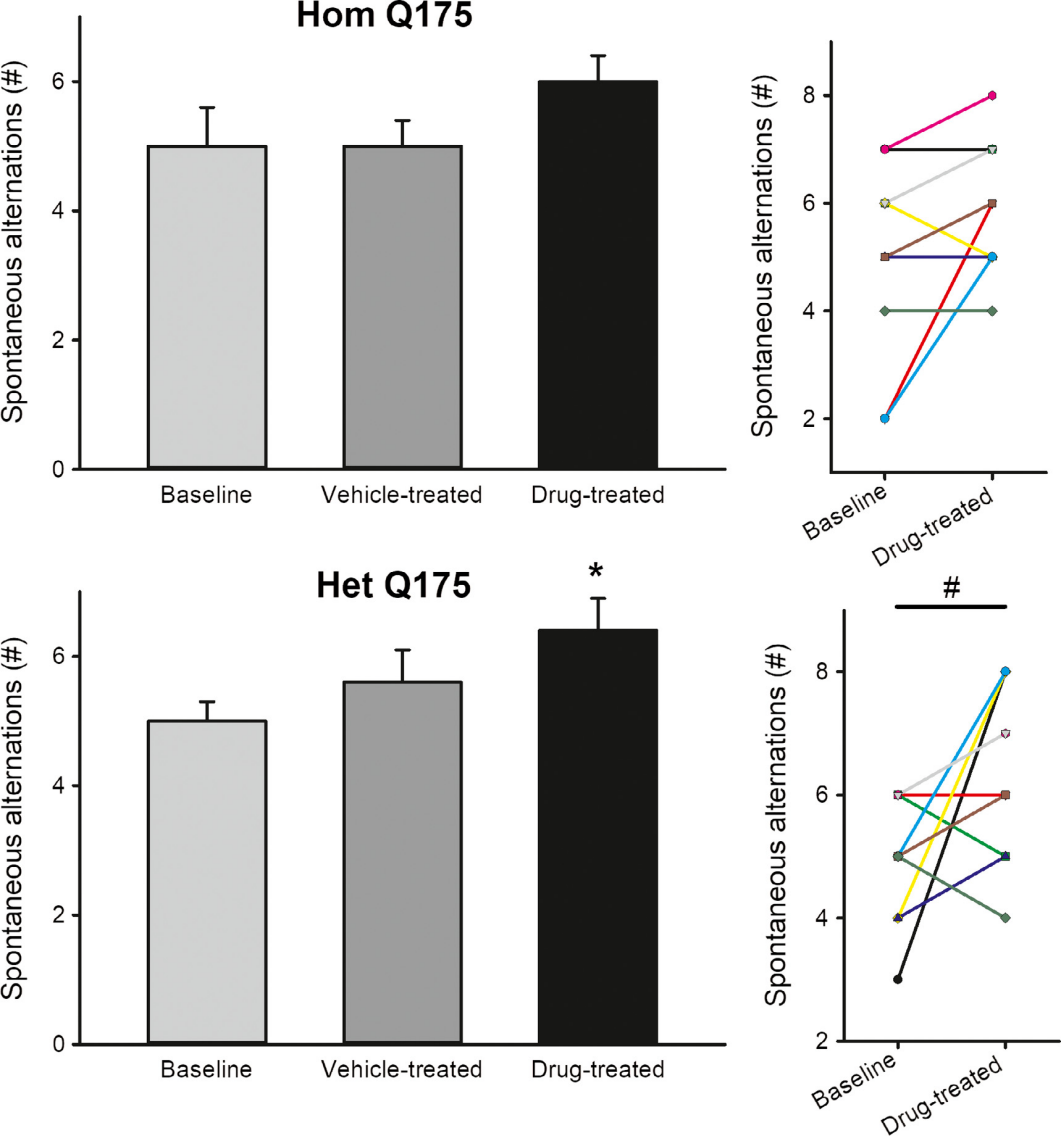
Chronic administration of GSK189254 improved cognitive performance as measured by the T‐maze in the Het Q175 mice. One‐way ANOVA was used to analyze the group values. Asterisks represent significant differences due to drug treatment compared to vehicle‐treated controls (*P *<* *0.05). A paired *t*‐test was used to compare the values from the same animals before and after treatment. Significance (*P *<* *0.05) shown by a # symbol.

In the TM test, the benefits of the drug were seen in the Het Q175 (Fig. 6). Compared to vehicle‐treated controls, drug treatment significantly increased spontaneous alternations in the Het (*H *=* *9.612, *P *=* *0.022) but not in the Hom Q175 (*F *=* *1.224, *P *=* *0.310). A comparison of the same animals before and after treatment (paired *t*‐test) also indicated that the drug treatment produced significant increases in the alternations in the Het (*t *= −2.753, *P *=* *0.022) but not the Hom (*t *= −2.123, *P *=* *0.0629) Q175 (Fig. 6). The results from the OF and TM tests indicate that the GSK189254 boosted performance on the tests without an increase in anxiety‐like behavior in the mutant lines.

### Chronic GSK189254 reduced behavioral despair

**Figure 7:**
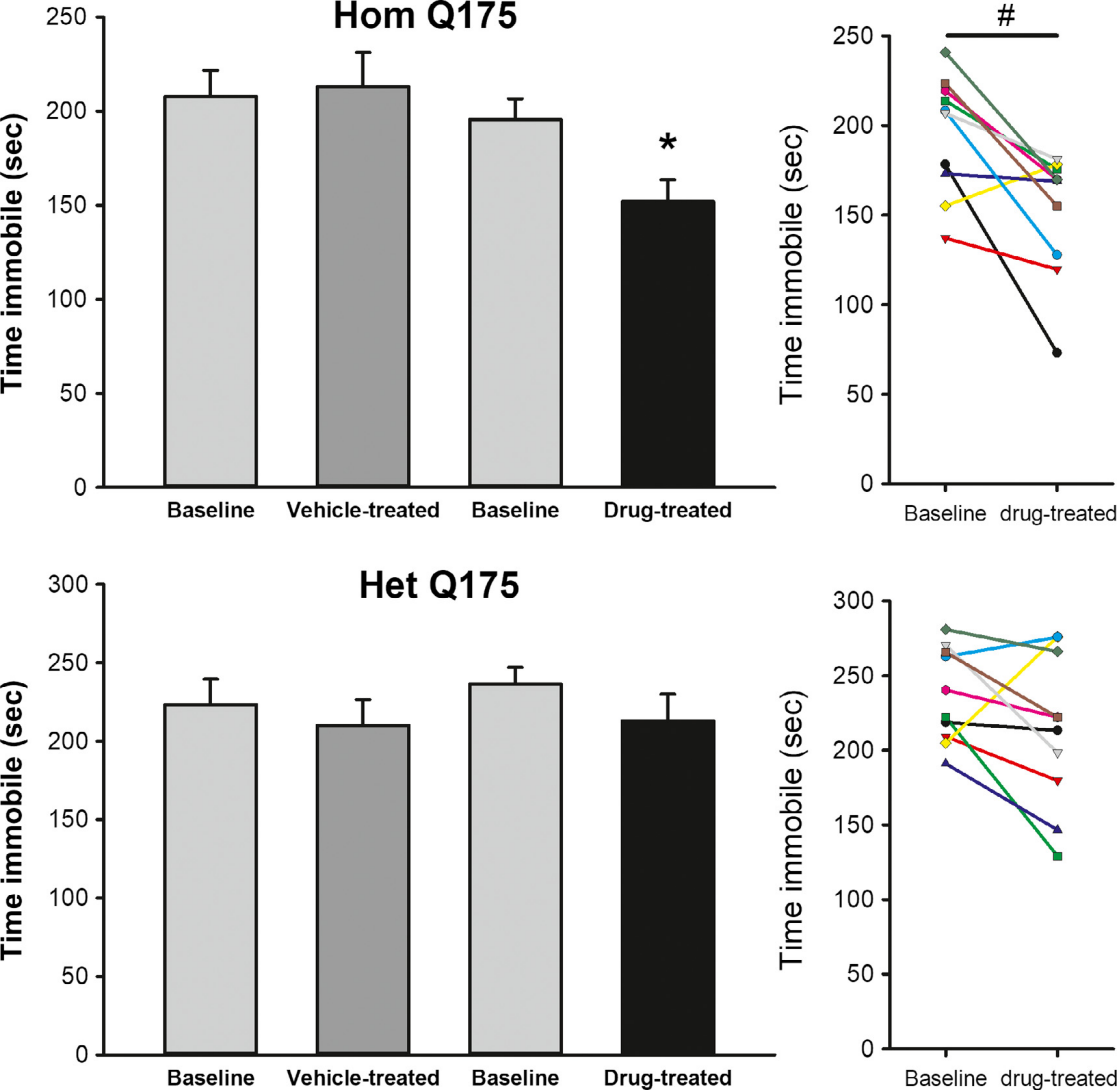
Chronic administration of GSK189254 improved affect as measured by the tail suspension test in Hom Q175 mice. One‐way ANOVA was used to analyze the group values. Asterisks represent significant differences due to drug treatment compared to vehicle‐treated controls (*P *<* *0.05). A paired *t*‐test was used to compare the immobility values from the same animals before and after treatment. Significance (*P *<* *0.05) shown by a # symbol.

The TS test is a commonly used measure of affect in mice, with time that the mouse is immobile as an indicator of behavioral despair. Compared to vehicle controls, chronic administration of GSK189254 significantly decreased the immobile time in the Hom (*H *=* *13.381, *P *=* *0.006) but not the Het (*F *=* *0.680, *P *=* *0.570) Q175 mice. A comparison of the same animals before and after treatment (paired *t*‐test) also indicated that the drug treatment produced significant decreases in immobility in the Hom (*t *=* *3.555, *P *=* *0.006) but not the Het (*t *=* *1.638, *P *=* *0.136) Q175 (Fig. 7).

To evaluate whether the H3R antagonist might increase aggressive behavior, we used RI test in which a stranger mouse is introduced into the home cage of a Q175 mouse. In the Hom Q175, half of the drug‐treated mice (5 of 10) exhibited some type of aggression against the stranger mouse in the test period (5 min), whereas none of the vehicle‐treated mice (0 of 10) exhibited aggressive behavior. In the Het Q175, none of the drug treated (0 of 10) nor any of the vehicle controls (0 out 9) exhibited any evidence of aggression during this same test.

### Chronic GSK189254 did not alter motor performance

Finally, we examined the impact of drug treatment on the CB test. Compared to vehicle controls, chronic administration of GSK189254 did not alter the number of errors made by either Hom (baseline: 3.8 ± 0.4; vehicle: 4.3 ± 0.6; drug: 4.4 ± 0.4; *F *=* *0.067, *P *=* *0.935) or Het (baseline: 3.9 ± 0.4; vehicle: 4.1 ± 0.7; drug: 3.8 ± 0.4; *F *=* *0.101, *P *=* *0.904) Q175. Similarly, there was no difference in the time (sec) that the mouse took to cross the beam in Hom (baseline: 14.8 ± 1.0; vehicle: 13.7 ± 0.5; drug: 15.6 ± 0.7; *F *=* *1.438, *P *=* *0.255) or Het (baseline: 16.0 ± 1.0; vehicle: 18.2 ± 1.6; drug: 16.9 ± 1.5; *F *=* *0.754, *P *=* *0.480). Thus, treatment with GSK189254 did not alter the performance on the CB test.

## Discussion

In this study, we sought to determine if the daily administration of the H3R antagonist/inverse agonist GSK189254 would improve nonmotor symptoms in the Q175 mouse model of HD. We administered the GSK189254 compound nightly to Hom and Het Q175 mice for 4 weeks starting at ages before the onset of motor symptoms and confirmed that the plasma levels of the drug were elevated to a therapeutic range. We demonstrate that daily treatment with GSK189254 improved the daily activity rhythm with increases in the strength of the rhythm as measured by power of the periodogram and decreases in cycle‐to‐cycle variability in activity onset. GSK189254 treatment also produced short‐term changes in sleep behavior but did not cause an overall change in the amount of sleep as measured over the 24‐h cycle. GSK189254 treatment improved performance of the HD mutant mice on exploratory behavior, cognitive performance and mood without increasing anxiety‐like behavior. The drug treatment did not alter motor performance and coordination as measured by the challenging beam test. Our findings suggest that drugs targeting the H3R system may benefit somnolence and vigilance in the management of HD.

A potential role for H3R antagonists in promoting wakefulness is supported by a number of previous studies. Centrally, HA is produced by TMN neurons and is thought to play a critical role in daily rhythms in arousal (Brown et al. 2001; Haas and Panula 2003; Lin et al. 2011) with peak release during the active cycle and relatively lower levels during sleep (Mochizuki et al. 1992; Prast et al. 1992). Like other monoamine neurotransmitters, HA has a specialized class of autoreceptors (the H3R) that serve as a synaptic negative feedback system. Activation of H3R results in the inhibition of HA synthesis (Gomez‐Ramirez et al. 2002) and release from histaminergic neurons (Arrang et al. 1983). Because the H3R are constitutively activated (Morisset et al. 2000; Takahashi et al. 2003), pharmacological agents that block H3Rs increase the release of HA. H3R are widely expressed in the mammalian brain, particularly in areas involved in cognitive processes and arousal, such as the cerebral cortex, hippocampus, basal ganglia, and hypothalamus (Martinez‐Mir et al. 1990; Pollard et al. 1993; Pillot et al. 2002). In cultured cortical neurons, activation of H3R results in the phosphorylation of the Akt/GSK‐3 beta pathway (Mariottini et al. 2009). Prior work indicates that H3R antagonists and inverse agonists promote wakefulness via increased HA release stimulating postsynaptic Histamine‐1 receptors (Lin et al. 1990; Barbier et al. 2004; Fox et al. 2005; Bonaventure et al. 2007). The drug used in this study (GSK189254) is a potent H3R antagonist (human H3R Ki = 0.2 nmol/L) which increased wakefulness, and improved performance of rats on a number of tests including passive avoidance, water maze, and object recognition at the same dose as used in this study (3.0 mg/kg) (Medhurst et al. 2007; Griebel et al. 2012). It is important to consider that besides increasing HA itself, GSK189254 increased acetylcholine, norepinephrine, and dopamine as measured by microdialysis (Medhurst et al. 2007). In rats, perfusion of the TMN with GSK189254 increased histamine release from the TMN and cortex, but not from the striatum or nucleus accumbens (Giannoni et al. 2010). Broadly speaking, H3R antagonists and inverse agonists have been found to promote wakefulness in a wide range of studies (review in Passani et al. 2004; Lin et al. 2011).

In this study, we administered the H3R antagonist nightly when HA levels would normally be rising. For reasons that could be due to differences in absorption or clearance, the drug levels in the serum were higher in the Hom Q175 than in the Het Q175. Behaviorally, the drug application also increased activity up to four hrs for the Hom Q175 and for at least 1 h for the Het Q175. Regardless, mice of both genotypes exhibited a strengthened activity rhythm with improved precision in activity onset. Additionally, the temporal pattern of sleep behavior was altered with reduced sleep immediately after drug treatment, yet the treatment did not alter the total amount of sleep within a daily cycle. Our observation that daily administration of the H3R antagonist improves the activity rhythm fits a significant body of evidence which indicates that HA is also a potent regulator of the circadian time‐keeping system in mammals. The mammalian circadian clock (SCN) receives a dense histaminergic input from the TMN (Watanabe et al. 1984) and expresses HA receptors (Michelsen et al. 2005; Kim et al. 2015) including the *Hrh3* gene (Allen Brain Atlas, Hrh3, exp. 73636034, probe RP_050825_02_B04 – coronal). A recent study also found that *Hrh3* expression varies rhythmically in the SCN with peak levels found in the late day (Pembroke et al. 2015). There is strong evidence that HA can phase‐shift the circadian neural activity rhythms in rodent SCN slices (Cote and Harrington 1993; Meyer et al. 1998; Biello 2009; Kim et al. 2015). Inhibition of HA synthesis disrupts the circadian rhythms of locomotor activity, sleep, and corticosterone release (Itowi et al. 1989, 1990, 1991) and histamine‐1 receptor knockout (KO) mice exhibit disruption of the circadian rhythms with decreased activity during the active phase (Inoue et al. 1996; Barbier and Bradbury 2007). More recent work has found that the H3R KO exhibits substantially reduced locomotor activity rhythms (Rozov et al. 2015) without a change in the peak/trough expression of core clock genes (Gondard et al. 2013; Rozov et al. 2015). Thus, there is good reason to expect that the daily application of GSK189254 would alter the phase of the underlying circadian rhythm as well as influence the circadian control of locomotor activity.

Selective H3R antagonists have been shown to improve performance in a diverse range of rodent cognition paradigms, including object recognition, olfactory recognition, water maze, radial maze, and passive avoidance, with most pronounced effects being observed in models where a cognitive deficit is present such as in aged animals (Hancock and Fox 2004; Witkin and Nelson 2004; Medhurst et al. 2007). Our data suggest that these benefits can be extended to at least the Q175 HD models. We found modest but significant benefits to performance of the HD model on exploratory behavior in the open field, T‐maze, and tail suspension tests. As measured by the amount of time that the mice spent in the center vs. periphery of the open field, anxiety‐like behavior was also unchanged. The only potential negative indication was that the Hom Q175 did show an increase in aggressive behavior which should be explored in future work.

H3R are strongly expressed in the cortico‐striatal circuits controlling motor behavior (Martinez‐Mir et al. 1990; Pollard et al. 1993). In vitro autoradiography of [^3^H]GSK189254 found strong binding in the rat cortex and striatum; however, treatment with 3.0 mg/kg GSK189254 did not induce Fos expression in the striatum although it did increase Fos in cortical regions (Medhurst et al. 2007). As measured by number of errors and time required for passage of the mice in the challenging beam test, there were no changes in motor performance in the drug‐treated group. The H3R antagonists are known to increase vigilance so improvement might be found with more demanding motor tasks.

H3R antagonists have entered clinical trials for Parkinson's disease and Alzheimer's disease (Lin et al. 2011). A recent search through the NIH clinical trials data base (Clinicaltrials.gov, March 2017) found several clinical trials for histamine H3R antagonists either ongoing or planned. At present, modafinil is widely used as a wake‐promoting agent (e.g., Bastuji and Jouvet 1988; Dell'Osso et al. 2014). Modafinil improves waking, but the narcoleptic episodes persist. At least one study has shown that modafinil amplifies the wake‐promoting and antinarcoleptic effects of the H3R antagonist pitolisant, suggesting a synergy that could be clinically useful (Lin et al. 2008). Such a simultaneous use of two wake‐promoting agents seemed to be well tolerated by the animals because no clear signs of CNS overexcitation or hyperactivity were noted. GSK189254 suppresses narcoleptic episodes in orexin KO mice and repeated dosing (like that used in this study) reinforced the benefits (Guo et al. 2009). Based on the preclinical data obtained in this study, H3R antagonists like GSK189254 would be a reasonable candidate as a cognitive enhancer for HD patients. In addition, a broad range of neurological and psychiatric disorders exhibit disruption in the sleep/wake cycle. These disruptions are likely to have several negative consequences for the patients. Based on our findings, the H3R antagonists/inverse agonists could also provide benefits as agents that strengthen the activity/rest cycle.

## Author Contributions

Participated in research design: Whittaker, Wang, Loh, Cachope, Colwell; Conducted experiments: Whittaker, Wang; Performed data analysis: Whittaker, Wang, Loh, Colwell; Contributed to writing of the manuscript: Whittaker, Colwell.

## Disclosures

None declared.
